# Construction and Validation of the Perceived Opportunity to Craft Scale

**DOI:** 10.3389/fpsyg.2017.00573

**Published:** 2017-04-12

**Authors:** Jessica van Wingerden, Irene M. W. Niks

**Affiliations:** ^1^Centre of Knowledge and Innovation, Schouten GlobalZaltbommel, Netherlands; ^2^Department of Work and Organizational Psychology, Institute of Psychology, Erasmus University RotterdamRotterdam, Netherlands

**Keywords:** perceived opportunity to craft, job crafting, job resources, work engagement, scale development, validation

## Abstract

We developed and validated a scale to measure employees’ perceived opportunity to craft (POC) in two separate studies conducted in the Netherlands (total *N* = 2329). POC is defined as employees’ perception of their opportunity to craft their job. In Study 1, the perceived opportunity to craft scale (POCS) was developed and tested for its factor structure and reliability in an explorative way. Study 2 consisted of confirmatory analyses of the factor structure and reliability of the scale as well as examination of the discriminant and criterion-related validity of the POCS. The results indicated that the scale consists of one dimension and could be reliably measured with five items. Evidence was found for the discriminant validity of the POCS. The scale also showed criterion-related validity when correlated with job crafting (+), job resources (autonomy +; opportunities for professional development +), work engagement (+), and the inactive construct cynicism (-). We discuss the implications of these findings for theory and practice.

## Introduction

Over the last decade, the pace of technological change has been accelerating. These advances have a significant impact on the jobs of employees all over the world, which may vary from significant job creation and job displacement to widening skill gaps (World Economic Forum, 2016). Not only must industries and organizations be responsive to change, but so must employees be proactive and take responsibility for staying connected to their job and work environment. One approach through which employees can work on an optimal fit between their (changing) job and their own preferences and skills is job crafting. Job crafting is defined as employees’ self-initiated change behaviors that aim to align their jobs with their own preferences, motives, and passions (Wrzesniewski and Dutton, 2001; see also Berg and Dutton, 2008). Because research has revealed that employees’ job crafting behavior is positively related to their well-being (Bakker et al., 2012) and work performance (Leana et al., 2009), researchers and organizations are interested in ways to stimulate job crafting.

Literature suggests that employees’ actual job crafting behavior may depend on the opportunities they perceive to do so (Wrzesniewski and Dutton, 2001; Wrzesniewski, 2003; van Wingerden et al., 2013). To empirically examine the suggested relation between employees’ perceived opportunities to craft and their job crafting behavior, a validated perceived opportunity to craft scale (POCS) is essential. Therefore, the central aim of the present two studies is to develop and validate a generic scale to measure employees’ perceived opportunity to craft (POC). After providing the theoretical background of POC, we will present the two studies. In Study 1, we developed the POCS and examined its psychometric properties (i.e., reliability and factorial validity). In Study 2, we cross-validated the factor structure of the POCS and examined its criterion-related and discriminant validity.

## Theoretical Background

### Job Crafting

In the beginning of the 21st century, Wrzesniewski and Dutton (2001) labeled the pro-active changes employees make to their jobs as “job crafting.” More specifically, job crafting was defined as employees’ self-initiated, proactive behavior aimed at aligning their jobs with their own preferences, motives, and passions. According to Wrzesniewski and Dutton (2001), employees can craft their job by changing their job tasks, their relationships at work and their perceptions about work. Although the concept of job crafting dates from 2001, employees’ proactive behavior was the subject of research decades earlier. Studies conducted in the eighties and nineties had already suggested that employees make self-initiated changes at work (Nicholson, 1984; Staw and Boettger, 1990) that may become accepted as part of employees’ contributions to the organization. In other words, these proactive changes may result in permanent changes in their job design (LePine and Van Dyne, 1998).

Because job crafting involves initiating changes in the design of the job, Tims et al. (2012) operationalized job crafting according to the types of job characteristics suggested by the Job Demands-Resources (JD-R) theory. Accordingly, every job consists of job demands and resources. Job demands are the physical, social, or organizational aspects of the job that require physical and/or cognitive engagement and that are associated with physical and psychological costs (Demerouti et al., 2001, p. 501). Examples of job demands are work pressure and complex assignments. Job resources are the physical, psychological, social, and organizational aspects of the job that help employees to achieve their work goals (Demerouti et al., 2001). Examples of job resources are opportunities for professional development, autonomy, and feedback.

According to Tims et al. (2012), job crafting consists of four dimensions: increasing social job resources (e.g., asking for feedback), increasing structural job resources (e.g., creating opportunities to participate in decision making), increasing challenging job demands (e.g., starting new projects), and decreasing hindering job demands (e.g., lowering the amount of work tasks). However, various recent studies have shown negative results of decreasing hindering job demands (e.g., Petrou et al., 2012, 2016; Tims et al., 2013; Gordon et al., 2015). Therefore, we will not include this dimension in the current study.

The job crafting approaches by Wrzesniewski and Dutton (2001) and by Tims et al. (2012) are similar in the sense that they both suggest that job crafting concerns employees’ self-initiated changes to optimize their work environment. The main difference, however, is that Tims et al. (2012) define job crafting as actual behavior, whereas Wrzesniewski and Dutton (2001) explicitly include a cognitive job crafting aspect as well. That is, they propose that employees can alter their view of work. For example, school janitors may reframe their job as creating an optimal learning environment for the students, as opposed to merely maintaining the school building. This may contribute to a more positive work experience. Thus, through job crafting, employees can optimize their work environment and work experience. This may affect both individual (e.g., well-being) and organizational outcomes (e.g., performance) (Bakker and Demerouti, 2014).

In the 15 years after publishing the article “Crafting a job: revisioning employees as active crafters of their work” (Wrzesniewski and Dutton, 2001), job crafting became a popular topic for researchers all over the world. Different studies confirmed the proposed positive relationship between job crafting on the one hand, and individual and organizational outcomes on the other (Leana et al., 2009; Tims et al., 2013; van Wingerden, 2016). More specifically, research revealed that employees’ job crafting behavior is positively related to their personal resources (van Wingerden et al., 2017), job resources (van den Heuvel et al., 2015), organizational commitment (Ghitulescu, 2007), basic need satisfaction (van Wingerden, 2016), work engagement (Bakker et al., 2012), and work performance (Leana et al., 2009). Because enhancing job crafting behavior among employees may result in improved organizational performance, job crafting has also become a subject of interest among senior management (e.g., Evans and Holmes, 2013).

### Job Crafting and Perceived Opportunity to Craft

Although job crafting concerns employees’ self-initiated actions to optimize their work environment, employees’ actual job crafting behavior may depend on the opportunities they perceive to do so (Wrzesniewski and Dutton, 2001; Wrzesniewski, 2003). POC can be defined as employees’ perception of their opportunity to craft their job. This perception may be influenced by internal and external factors. For instance, job crafting has been shown to be positively related with proactive personality (Bakker et al., 2012) and approach temperament (Bipp and Demerouti, 2015) (both internal factors). A recent qualitative job crafting intervention study (van Wingerden et al., 2013) among teachers confirmed the assumption that employees’ perceived opportunities to craft may determine whether they will proactively craft their job. Participants of the job crafting intervention who reported that they did *not* succeed in crafting their job stated that they did not perceive opportunities to do so. In this study, however, reasons for not perceiving these opportunities as reported by the participants consisted of external attributions. That is, these employees felt that changes in their work were restricted by managers, behavioral patterns on the job, the organization, and the Ministry of Education (external factors). In contrast, their colleagues who did perceive that they had opportunities to craft their job reported that they successfully made self-initiated changes to their work environment.

To further examine the concept and antecedents of POC, a validated measurement instrument is necessary. Developing a scale to quantitatively measure POC may help to gain a deeper understanding of employees’ job crafting behavior. In addition, it may contribute to discovering how organizations and/or senior management can influence and stimulate job crafting behavior among their employees.

## Study 1: Scale Development and Explorative Test

The goal of the first study is to develop and test a generic scale that can be used to quantitatively measure POC. POC reflects an overall perception of the extent to which employees can influence their work environment. Therefore, we expect a one-dimensional structure for the POCS (*Hypothesis 1*). Although POC is conceptually appealing, there has been little systematic effort to empirically examine the concept. In developing a scale that is applicable to different occupations, we aim to encourage more empirical research on POC. We first describe the process of constructing the scale and then present the exploratory results of the scale’s factorial structure and reliability.

### Methods

#### Scale Construction

To develop an item pool, we first studied the literature and available measures of POC. Then we selected and developed appropriate items taking the following criteria into account. First, items had to reflect employees’ perceptions of their opportunities to craft rather than their actual crafting behavior. Second, specific work context terminology was avoided so that the POCS would be applicable to all work contexts. Because our literature study revealed that there were no available measures of POC, we developed a scale based on the available job crafting literature.

Initially, we constructed a pool of 12 items. All items were formulated as declarative statements following the stem “The following statements aim to tap into your perceptions of the opportunities you have to change aspects of your job.” Responses were made on a 7-point frequency scale ranging from 1 (totally disagree) to 7 (totally agree). Before we collected data on the POCS, we first had two discussion sessions with a panel of four Work and Organizational psychologists about the proposed definition of POC and the constructed items. To test the content validity of items (I-CVI) and the overall scale (S-CVI), we followed the procedure suggested by Lynn (1986). Each panel member evaluated two different criteria, that is, the clarity of language and the relevance of the question for the construct of POC. Clarity of language used in the questionnaire was assessed through the question: “To what extent do you believe that this item is understandable across different occupational populations?” The relevance of the question for the POCS was assessed through the question: “To what extent do you believe that this item is relevant to assess the perception of opportunities to craft one’s job in the workplace?” Each panel member rated these two aspects of all items using a 4-point Likert scale with 1 = irrelevant; 2 = somewhat relevant; 3 = quite relevant, and 4 = extremely relevant. The I-CVI was computed as the number of experts evaluating the items as quite relevant (3) or extremely relevant (4), divided by the total number of experts. According to Lynn (1986), the I-CVI should be 1.00 when there are five or fewer experts, which is the case in our study. Therefore only items reporting a total agreement between the work and organizational psychologists for both the above-mentioned criteria were included in the scale. As a result, six items were maintained. The Work and Organizational psychologists had worked on average 5 years in both research and practice. To examine whether the newly constructed scale does indeed consist of one dimension, we explored the factor structure and reliability of the scale.

#### Participants and Procedure

Education professionals from a Dutch institution for special education were invited to participate in the study by filling out an online employee engagement survey. This survey contained several constructs related to the work situation, work environment, and employee outcomes. The managing director introduced the survey to 976 education professionals via an email containing a link to the online survey, which was available for a period of 3 weeks. The procedure and goals of the study were explained, while also addressing the anonymity of the data. After 3 weeks 733 teachers had responded, making a response rate of 75.1%. The sample consisted of 637 female (86.9%) and 96 male teachers (13.1%), which is representative for the occupational group. The mean age of the participants was 46 years (*SD* = 10.91). The education level of the sample was relatively high, as 82.6% possessed at least a bachelor’s degree. Data has been collected in accordance with the ethical guidelines of the American Psychological Association and the Dutch Association of Psychologists. As such, (1) participation was completely voluntary, (2) data collection through a self-report survey is exempted from an institutional ethics committee’s approval, and (3) the respondents did not receive any monetary compensation for their contribution. Informed consent was given by clicking on the “Finish” button on the last page of the survey.

### Results

#### Exploratory Factor Analysis

We used principal factor analysis (maximum likelihood) with oblique rotation in SPSS to examine the factor structure of the six items. As a criterion to retain factors, those factors that had an eigenvalue >1 were retained. In addition, we retained items that loaded 0.35 or higher on the expected factor (Floyd and Widaman, 1995; Costello and Osborne, 2005). On the basis of these criteria, all six items were retained.

The results showed that POC loads on one factor, which contains six items. The items, item means, standard deviations, Cronbach’s alpha, and factor loadings are presented in **Table 1**. The factor (eigenvalue = 3.49), which explained 58.16% of the variance, is formed by the six items for POC. The POCS has a good reliability with a Cronbach’s alpha of 0.85, which is amply above the required 0.70 (Nunnally and Bernstein, 1994). The results of Study 1 provide conceptual support for the hypothesized one-factor structure (*Hypothesis 1*). It is important to replicate this factor structure in other, independent samples to rule out that the one-factor structure is due to specific characteristics of the current sample. Therefore, the aim of Study 2 is to cross-validate the factor structure of the POC and, subsequently, to examine its criterion-related and discriminant validity.

**Table 1:** Study 1: items, means, standard deviations, Cronbach’s alpha, and factor loadings of the POCS (*N* = 733).

	Item wording	*M*	*SD*	α	Factor loading
	*Perceived opportunity to craft*			0.85	
1	At work I have the opportunity to vary the type of tasks I carry out	4.98	1.24		0.71
2	At work I have the opportunity to adjust the number of tasks I carry out	4.41	1.45		0.73
3	At work I have the opportunity to choose who I want to work with^∗^	3.96	1.53		0.72
4	At work I have the opportunity to vary my contacts with other people	4.56	1.27		0.75
5	At work I have the opportunity to take on new activities and challenges	4.21	1.35		0.84
6	At work I have the opportunity to change the meaning of my role	4.23	1.32		0.82

*Items were translated in English. ^∗^This item was deleted from the scale based on results of Study 2.*

## Study 2: Confirmatory Factor Analysis, Reliability, and Validity of the POCS

In this study, we first examine whether mean values on the POCS differ between industries. Employees’ perceived opportunities to craft may for example be lower in industries with work environments that are characterized by strict protocol and rules (e.g., health care), than in industries with work environments that are characterized by autonomy and creativity (e.g., professional services). We therefore hypothesize that means on the POCS differ between industries (*Hypothesis 2*). In addition, we examine whether the one-factor structure can be reliably replicated in two new samples using confirmatory factor analysis. We hypothesize that the one-factor model will show a good fit to the data in both samples (*Hypothesis 3*). Further, we test the robustness of the scale in these samples with an invariance test. Finally, we examine the criterion-related and discriminant validity of the POCS. To our knowledge, there is no general POC measure yet. We therefore relate the POCS to theoretically related constructs. First, we expect a positive relationship between POC and job crafting (*Hypothesis 4*). Second, we expect POC to be positively related to job resources (i.e., autonomy and opportunities for professional development) because job resources are elements of employees’ perceptions of the work environment that are directly related to job crafting (*Hypothesis 5*).

We also aim to explore how POC is related to favorable and unfavorable work attitudes. In this light, we will examine its associations with work engagement and cynicism, which is a sub-dimension of burnout. We expect a positive relationship between POC and work engagement, as both concepts reflect a favorable perception of the work environment (*Hypothesis 6*). In contrast, cynicism may be negatively associated with POC. Cynicism is defined as a distant or indifferent attitude toward work (Schaufeli and Bakker, 2004). Cynical employees may withdraw themselves from work because they are less involved in the organization (Richardsen et al., 2006), which may negatively influence their perception of the work environment. We therefore hypothesize a negative relationship between POC and cynicism (*Hypothesis 7*).

### Methods

#### Procedure and Participants

We collected data in two new Dutch samples. For the first sample, data were collected using an online survey. The study was announced on a well-known Dutch career development website as well as through various social media channels. Respondents were invited to participate on a voluntary basis and directed to the survey through an online link. Also for this study, ethical guidelines of the American Psychological Association and the Dutch Association of Psychologists were followed. Informed consent was again given by clicking on the “Finish” button on the last page of the survey. The survey was in Dutch and available for 3 weeks. In total, 612 employees filled out the survey. A majority of the sample was female (61.6%) and the mean age of the participants was 46 years (*SD* = 10.1). Most participants (78.1%) reported that they possessed at least a bachelor’s degree. Various sectors were represented, with most participants working in health care (16.8%), professional services (13.2%), the public sector (14.4%), education (11.8%), industry (8.3%), and information technology (8.2%).

The second sample was recruited following the same procedure as the first sample. In this sample of 984 Dutch employees, 65% was female. The mean age of the participants was 44.7 years (*SD* = 10.7) and a large proportion of the participants possessed at least a bachelor’s degree (79.5%). Most participants worked in professional services (19.8%), health care (13.6%), the public sector (12.9%), education (11.8%), industry (8.5%), and information technology (7.2%). In sum, the two samples resemble each other regarding gender, age, educational level, and sector.

#### Measures

*Perceived opportunity to craft* was measured with the six items reported in Study 1 (see **Table 1**).

*Autonomy* was assessed with a 3-item scale, based on Karasek’s (1985) job content instrument. A sample item is “I can decide myself how I execute my work.” All items were scored on a 5-point scale where the scale ranged from 1 (never) to 5 (always).

*Opportunities for professional development* were assessed with three items from the scale of Bakker et al. (2003). A sample item is “I have sufficient possibilities to develop myself at work.” All items were scored on a 5-point scale ranging from 1 (totally disagree) to 5 (totally agree).

*Job crafting* was measured using three subscales of the job crafting questionnaire developed by Tims et al. (2012). Of each subscale, four items were included and scored on a 5-point scale ranging from 1 (*never*) to 5 (*very often*). Examples are: “I ask colleagues for advice” (increasing social job resources), “I regularly take on extra tasks even though I do not receive extra salary for them” (increasing challenging job demands), and “I try to learn new things at work” (increasing structural job resources).

*Work engagement* was measured with the 9-item Utrecht Work Engagement Scale (UWES; Schaufeli et al., 2006). The instrument consists of three subscales to assess vigor, dedication, and absorption. Examples for each subscale are “At work, I am bursting with energy” (vigor), “I am enthusiastic about my job” (dedication), and “I am immersed in my work” (absorption). Participants could respond to these items using a 7-point Likert-scale ranging from 0 (*never*) to 6 (*always*).

*Cynicism* was measured with four items pertaining to the subscale of the Dutch version (Schaufeli and Van Dierendonck, 2000) of the Maslach Burnout Inventory—General Survey (Schaufeli et al., 1996). The items were scored on a 7-point Likert-scale ranging from 0 (*never*) to 6 (*always*). An example item is “I doubt the significance of my work.”

### Results

#### Preliminary Analysis

First, we analyzed the overall mean scores and standard deviation of POC using the combined samples, resulting in *M* = 4.66 (*SD* = 1.17). As mean values on the POCS may differ between industries (*Hypothesis 2*), we also analyzed the scores for each industry: professional services (*M* = 4.89), health care (*M* = 4.50), the public sector (*M* = 4.55), education (*M* = 4.70), industry (*M* = 4.76), and information technology (*M* = 4.83). Analyses revealed that the sector professional services scored significantly higher than the public sector and health care (*p* < 0.05). These outcomes confirm Hypothesis 2. Reliabilities and correlations of all measures in the two samples are depicted in **Table 2**.

**Table 2:** Zero-order correlations and Cronbach’s alphas among all study variables in samples 1 and 2.

	Variable	1	2	3	4	5	6	7	8
1.	POC	(0.91/0.88)	0.43ˆ**	0.62ˆ**	0.64ˆ**	0.49ˆ**	0.52ˆ**	0.42ˆ**	–0.48ˆ**
2.	Job crafting	0.53ˆ**	(0.88/0.85)	35^∗∗^	0.32ˆ**	0.37ˆ**	0.38ˆ**	0.38ˆ**	–0.16ˆ**
3.	Autonomy	0.67ˆ**	0.40ˆ**	(0.86/0.82)	0.48ˆ**	0.44ˆ**	0.46ˆ**	0.39ˆ**	–0.38ˆ**
4.	Development opportunities	0.66ˆ**	0.38ˆ**	0.54ˆ**	(0.90/0.89)	0.48ˆ**	0.62ˆ**	0.48ˆ**	–0.57ˆ**
5.	Vigor	0.50ˆ**	0.49ˆ**	0.43ˆ**	0.53ˆ**	(0.86/0.85)	0.77ˆ**	0.67ˆ**	–0.62ˆ**
6.	Dedication	0.54ˆ**	0.51ˆ**	0.48ˆ**	0.64ˆ**	0.84ˆ**	(0.92/0.89)	0.77ˆ**	–0.70ˆ**
7.	Absorption	0.43ˆ**	0.49ˆ**	0.41ˆ**	0.49ˆ**	0.74ˆ**	0.79ˆ**	(0.77/0.74)	–0.49ˆ**
8.	Cynicism	–0.47ˆ**	–0.30ˆ**	–0.42ˆ**	–0.57ˆ**	–0.66ˆ**	–0.71ˆ**	–0.50ˆ**	(0.86/0.83)

*Correlations below the diagonal are sample 1 correlations (*N* = 612). Correlations above the diagonal are sample 2 correlations (*N* = 984). The values on the diagonal represent Cronbach’s alpha for samples 1 and 2, respectively. Correlations are based on the 5-item version of the POC scale. ^∗∗^*p* < 0.01.*

#### Confirmatory Factor Analysis

To test whether the one-factor solution also fits best in these two new samples (*Hypothesis 3*), we used multigroup confirmatory factor analysis within the AMOS software package (Arbuckle, 2005). With multigroup analysis it is possible to test the same model in two separate samples simultaneously. The results of this analysis yields one set of fit statistics for overall model fit (Byrne, 2004).

To assess model fit, we used five indices: χ^2^/*df* ratio, the Tucker–Lewis Index (TLI), the Comparative Fit Index (CFI; Bentler, 1990), the Incremental Fit Index (IFI), and the Root Mean Square Error of Approximation (RMSEA; Browne and Cudeck, 1993). With respect to the χ^2^/*df* ratio, values that are less than three generally indicate good model fit (Kline, 1998). Values of 0.90 and over (for TLI, CFI, and IFI) or 0.08 and under (for RMSEA) indicate acceptable fit (Byrne, 2001).

The results of the multigroup analysis regarding the goodness-of-fit indices of the one-factor solution are presented in the upper part of **Table 3**. The results revealed that the one-factor model consisting of six items fit the data inadequately (Model A). The factor loadings of the items were acceptable, ranging from 0.71 to 0.80. However, modification indices pointed toward a high covariance between the error terms of items 1 and 2 (“At work I have the opportunity to vary the type of tasks I carry out” and “At work I have the opportunity to adjust the number of tasks I carry out,” respectively). Including this covariance in the model (Model B) yielded a better, yet still unsatisfactory model fit (Δχ*^2^* = 602.48, Δ*df* = 2, *p* < 0.001), which seemed attributable to a suboptimal factor loading and item covariance of item 3 (“At work I have the opportunity to choose who I want to work with”). Closer inspection of this item raised the issue that in most jobs it is not realistic to choose who you want to work with, as co-workers are usually part of a formal work structure. Removing this item from the model indeed resulted in a substantially and significantly better model fit (Model C; Δχ^2^ = 239.73, Δ*df* = 8, *p* < 0.001). In addition, the goodness-of-fit indices of the model were (approximately) 0.99 and RMSEA was small (0.03) which support the acceptability of the fit (Bollen, 1989). The χ^2^/*df* ratio was also smaller than 3 for the one-factor model consisting of five items, indicating a good fit. Moreover, all items loaded significantly on the latent variables, with coefficients ranging from 0.59 to 0.88 (all *p*s < 0.001). Hence, a one-factor model based on five items adequately represented the observed data (*Hypothesis 3*), which is consistent with the results of Study 1.

**Table 3:** Multigroup confirmatory factor analysis and invariance test of the POC scale (*N* = 612 and *N* = 984).

Model	χ^2^	*df*	χ^2^*/df*	CFI	TLI	IFI	RMSEA
One-factor model: Model A	864.85	18	48.05	0.86	0.76	0.86	0.17
One-factor model: Model B	262.37	16	16.40	0.96	0.92	0.96	0.10
One-factor model: Model C	22.64	8	2.83	0.99	0.99	0.99	0.03
Invariance test						
Model 1 (default model)	22.64	8	2.83	0.99	0.99	0.99	0.03
Model 2 (fully constrained)	48.75	19	2.57	0.99	0.99	0.99	0.03
Model 3 (factor loadings constrained)	28.55	12	2.40	0.99	0.99	0.99	0.03
Model 4 (factor loadings and factor variance constrained)	32.57	13	2.51	0.99	0.99	0.99	0.03
Model 5 (final model)	36.88	16	2.31	0.99	0.99	0.99	0.03

#### Invariance Test

Invariance of the POCS in the two samples was also tested through multigroup analysis (Byrne, 2004). The starting point was a default model (Model 1 in **Table 3**) in which all parameters were estimated simultaneously without cross-group constraints. This model yielded a χ^2^ of 22.64 with 8 degrees of freedom, which served as the baseline value for subsequent model comparison. We then tested whether a fully constrained model (Model 2) was invariant across both samples. That is, all factor loadings, the factor variance, and all error (co)variances were constrained to be equal in both groups. Model 2 showed a significant difference with the default model (Δχ^2^ = 26.11, Δ*df* = 11, *p* < 0.01), indicating that some equality constraints did not hold across both samples (Byrne, 2004). We therefore proceeded to test in smaller steps for invariance.

First, we tested whether factor loadings were invariant across both groups. The fit of the model with constrained factor loadings (Model 3) did not show any improvement compared to the default model (Δχ^2^ = 5.91, *Δdf* = 4, *not significant*), implying that factor loadings were invariant. Next, we also constrained the factor variance (Model 4). This model was also as good as the unconstrained model, indicating that factor variance was invariant across groups (Δχ^2^ = 9.93, Δ*df* = 5, *not significant*). From these results it can be inferred that the remaining parameters, the error (co)variances, were not invariant. We then constrained the error variances and error covariance one by one, only adding constraints after they had proven to be invariant. The results indicated that only two out of five error variances as well as the error covariance were variant across groups. The model fit without these variant parameters (Model 5) was as good as the default model (Δχ^2^ = 14.23, Δ*df* = 8, *not significant*).

The results of the overall analysis are very satisfactory, given that we performed a highly stringent invariance test (Byrne, 2004). The fact that we only found cross-group variance in part of the error terms provides support for the invariance of the POCS.

#### Criterion-Related and Discriminant Validity

Before assessing the criterion-related validity of the POCS, we examined the potential overlap between POC, job crafting, job autonomy, opportunities for professional development, cynicism, and work engagement. We used multigroup confirmatory factor analyses to compare two-factor models (i.e., differentiating POC from each of the criterion-related variables) to one-factor models (i.e., modeling criterion-related variables as a latent factor together with POC). The results showed a better fit to the data for each of the two-factor models than the alternative one-factor models (see **Table 4**), indicating that the POCS can be empirically distinguished from the criterion-related variables.

**Table 4:** Multigroup confirmatory factor analyses for POC in combination with criterion-related study variables (*N* = 612 and *N* = 984).

Variables	Model	χ^2^	*df*	χ^2^*/df*	CFI	TLI	IFI	RMSEA	Δχ^2^	Δ*df*	*p*-value
POC and job crafting (JC): increasing structural job resources	Model 1: two factors	437.85	51	8.59	0.95	0.93	0.95	0.07			
	Model 2: one factor	1737.15	52	33.41	0.78	0.70	0.78	0.14	1299.29	1	0.000
POC and JC: increasing social job resources	Model 1: two factors	345.57	51	6.78	0.96	0.94	0.96	0.06			
	Model 2: one factor	1659.88	52	31.92	0.77	0.68	0.77	0.14	1314.31	1	0.000
POC and JC: increasing challenging demands	Model 1: two factors	155.20	51	3.04	0.99	0.98	0.99	0.04			
	Model 2: one factor	1662.01	52	31.96	0.78	0.69	0.78	0.14	1506.81	1	0.000
POC and autonomy	Model 1: two factors	310.44	37	8.84	0.96	0.95	0.96	0.07			
	Model 2: one factor	1247.45	38	32.83	0.85	0.78	0.85	0.14	937.02	1	0.000
POC and development opportunities	Model 1: two factors	82.01	37	2.27	0.99	0.99	0.99	0.03			
	Model 2: one factor	1069.43	38	28.14	0.88	0.83	0.88	0.13	987.42	1	0.000
POC and vigor	Model 1: two factors	110.43	37	2.99	0.99	0.99	0.99	0.04			
	Model 2: one factor	1662.77	38	43.76	0.79	0.69	0.79	0.16	1552.35	1	0.000
POC and dedication	Model 1: two factors	135.79	37	3.67	0.99	0.98	0.99	0.04			
	Model 2: one factor	1988.03	38	52.32	0.77	0.67	0.77	0.18	1852.24	1	0.000
POC and absorption	Model 1: two factors	71.46	37	1.93	0.99	0.99	0.99	0.02			
	Model 2: one factor	856.31	38	22.54	0.87	0.81	0.87	0.12	784.86	1	0.000
POC and cynicism	Model 1: two factors	189.00	51	3.71	0.98	0.97	0.98	0.04			
	Model 2: one factor	1789.84	52	34.42	0.78	0.70	0.78	0.15	1600.84	1	0.000

Also, on a most stringent test of discriminant validity we conducted a Fornell–Larcker test (Fornell and Larcker, 1981). The outcomes of this test showed support for discriminant validity of the POCS. That is, the square root of the average variance extracted for the POCS (i.e., 0.77) was greater than the inter-construct correlations between the different factors (i.e., ranging from 0.37 to 0.65). **Table 5** depicts the overall factor loadings, the composite reliability, and average variance extracted of the POCS.

**Table 5:** Study 2: factor loadings, composite reliability, and average variance extracted of the POCS (*N* = 1596).

Items	Factor loading	CR	AVE
*Perceived opportunity to craft*		0.85	0.59
At work I have the opportunity to vary the type of tasks I carry out	0.66		
At work I have the opportunity to adjust the number of tasks I carry out	0.63		
At work I have the opportunity to vary my contacts with other people	0.78		
At work I have the opportunity to take on new activities and challenges	0.88		
At work I have the opportunity to change the meaning of my role	0.85		

*CR, composite reliability; AVE, average variance extracted.*

The correlations between POC and the criterion-related variables in samples 1 and 2 are depicted in **Table 2**, respectively. As expected, POC was positively associated with job crafting (*Hypothesis 4*) as well as with job autonomy and opportunities for professional development (*Hypothesis 5*). Further, in line with *Hypothesis 6*, POC was also positively related to work engagement. Finally, as expected, the construct was negatively associated with cynicism (*Hypothesis 7*).

### Discussion Studies 1 and 2

The goal of the first study was to develop and test a generic scale that can be used to quantitatively measure POC. The results of Study 1 provide conceptual support for the hypothesized one-factor structure. The first aim of Study 2 was to examine whether the one-factor solution of the POCS in Study 1 could be reliably replicated with two new samples. Using multigroup confirmatory factor analyses we found that after removing one of the initial items, the one-factor model fit the data very well. The second aim of Study 2 was to test the robustness of the POCS by performing an invariance test. Our findings showed high invariance of the POCS, thereby providing support for the scale’s robustness. The third aim of this study was to examine the criterion-related and discriminant validity of the POCS. POC was found to be significantly related to job crafting, job resources, work engagement and cynicism in predictable ways. That is, in both samples all correlations between POC and the criterion-related variables were positive, except for a negative relation between POC and cynicism. These findings provide evidence for the criterion-related validity of the scale. In addition, confirmatory factor analyses demonstrated that POC can be empirically distinguished from job crafting, job resources, work engagement, and cynicism. Thus, the results indicated that POC and the criterion-related measures are related yet distinct constructs.

## General Discussion

Literature suggests that employees’ actual job crafting behavior may depend on their perceived opportunities to craft their jobs (Wrzesniewski and Dutton, 2001; Wrzesniewski, 2003; van Wingerden et al., 2013). However, until now no valid measure has been available to empirically examine the concept of POC. The newly developed and validated POCS may help researchers to gain more insight into the concept of job crafting, as well as its antecedents and consequences.

### The Perceived Opportunity to Craft Scale

Our results across three samples with 2329 employees in total provided good support for the psychometric properties of the POCS. In both studies, the scale demonstrated a one-factor structure. Also, our studies showed that POC on the one hand, and job crafting, job resources, work engagement, and cynicism on the other, are theoretically and empirically related yet distinctive constructs. These findings provide support for the added value of the POCS to the existing literature on job crafting.

### Limitations and Suggestions for Further Research

Some limitations need to be acknowledged. First, our findings support the criterion-related validity of the POCS by means of cross-sectional associations based on self-report. This type of data does not allow us to make causal inferences. However, we do expect a causal relation between POC and actual job crafting behavior, in the sense that POC may be a precondition. Future research may further examine causal relationships between POC and possible antecedents (e.g., job resources) and consequences (e.g., job crafting behavior), for instance, with longitudinal or daily diary study designs. It is plausible, for example, that high levels of job autonomy directly contribute to the POC. In turn, POC may be a precursor of actual job crafting behavior. To diminish potential common method bias (Podsakoff et al., 2003) it may also be interesting to include different types of criterion measures such as peer ratings of job crafting behavior and work engagement (cf. Tims et al., 2012).

A second limitation may be the relatively small number of items that were used to construct the POCS. A larger pool of items may have provided more room to explore the internal factor structure of the scale. However, as the construct of POC is rather narrow and straightforward and short measures are desirable for practical reasons, a limited number of items seemed appropriate (cf. Wanous et al., 1997; Abdel-Khalek, 2006). In fact, the current 5-item scale showed satisfactory psychometric properties and, as such, provides a reliable and valid way to measure POC.

Third, the present studies included one organization-specific sample as well as two heterogeneous convenience samples. A strength is that the samples included employees from a variety of sectors (e.g., health care, education, and professional services), implying that the POCS may be applicable to a wide range of occupations. Nevertheless, all participants across the three samples were Dutch. Future research may further add to the generalizability of the findings by validating the scale in other languages.

### Implications for Organizational Research

Our results indicate that POC has implications for job crafting behavior as well as for positive and negative employee outcomes. Literature shows that job crafting behavior is positively related to organizational commitment, work engagement, and performance. It is conceivable, however, that solely focusing on employees’ job crafting behavior may be ineffective when their POC is low. Therefore, the POCS may be important to business and HR from both a research and practical perspective. Insights in employees’ POC may be a starting point to understand and positively influence this perception. In turn, this may positively influence employee and organizational outcomes. As POC may be a precondition for actual job crafting behavior, managers should be aware of their potential influence on this perception. Employees’ POC may be facilitated or supported by management. For example, providing employees with autonomy, opportunities for professional development, and (positive) feedback on their job crafting actions may positively affect their perception of their opportunities to craft (see also Wrzesniewski, 2003). Also, it may be that the perception of (not) having opportunities to craft itself directly affects work attitudes. These issues remain to be investigated. We hope to stimulate and encourage researchers and practitioners in the field of job crafting to take on this challenge by providing a generic, reliable, and valid scale to measure POC. As such, the POCS may contribute to assessing and enhancing the impact of job crafting behavior in organizations.

## Author Contributions

JVW designed the study and collected the data. IN analyzed the data. JVW and IN both drafted the manuscript.

## Conflict of Interest Statement

The authors declare that the research was conducted in the absence of any commercial or financial relationships that could be construed as a potential conflict of interest.
